# Natural frequencies of neural activities and cognitions may serve as precise targets of rhythmic interventions to the aging brain

**DOI:** 10.3389/fnagi.2022.988193

**Published:** 2022-09-12

**Authors:** Jingwen Qiao, Yifeng Wang, Shouyan Wang

**Affiliations:** ^1^Academy for Engineering and Technology, Fudan University, Shanghai, China; ^2^Institute of Brain and Psychological Sciences, Sichuan Normal University, Chengdu, China; ^3^Institute of Science and Technology for Brain-Inspired Intelligence, Fudan University, Shanghai, China

**Keywords:** frequency shift, natural frequency, neurodegenerative diseases, rhythmic brain stimulation, temporal dedifferentiation

## Abstract

Rhythmic neural activities are critical to the efficiency of regulatory procedures in brain functions. However, brain functions usually decline in aging as accompanied by frequency shift and temporal dedifferentiation of neural activities. Considering the strong oscillations and long-lasting after-effects induced by rhythmic brain stimulations, we suggest that non-invasive rhythmic brain stimulation technique may help restore the natural frequencies of neural activities in aging to that in younger and healthy brains. Although with tremendous work to do, this technique offers great opportunities for the restoration of normal brain functions in aging, or even in those suffering from neurodegenerative diseases and neuropsychiatric disorders.

## Introduction

Rhythmic neural activities are the basic characteristic of brain function (Buzsáki, 2006). In response to various cognitive demands, the human brain functions across multiple frequencies (Soltani et al., 2021) with distinct mechanisms (Brookes et al., 2016) or multi-layer functional networks (Sasai et al., 2020). Intrinsic frequencies have been stressed for local neural activities (Murray et al., 2014), neural circuits (Rosanova et al., 2009), as well as cognitive functions (Palva and Palva, 2018). Neural activities at intrinsic frequencies form spectral fingerprints of brain functions (Siegel et al., 2012). Throughout the human lifespan, frequencies of these neural activities, however, are constantly changing, as presented in the form of the shift of frequency between adjacent frequency bands and/or temporal dedifferentiation among multiple bands (Alcauter et al., 2015; Yang et al., 2018; Ao et al., 2022). For instance, the individual alpha peak frequency slowed from 10 Hz at the age of 20 years (i.e., younger age) to 8.8 Hz at the age of 70 years (i.e., older age) and from 9.9 Hz in healthy adults aged 18–60 years old to 9.4 Hz in patients with schizophrenia of the same age range (Scally et al., 2018; Ramsay et al., 2021). These altered frequency characteristics due to aging and/or age-related conditions are closely associated with changes in brain functions and behavioral performances, such as cognitive and motor functions, the control of standing and walking (He et al., 2010; Assenza et al., 2017). In other words, such altered frequency characteristics may interfere with neural activities that are related to cognitive and behavioral performances, leading to their non-optimal (less efficient) neural control (Klimesch, 2018; Wolinski et al., 2018).

This may be a potential explanation for the findings that though many cognitive training strategies can help improve cognitive performances in relatively older adults, no benefits for the prevention of age-related cognitive decline have been observed (Butler et al., 2018), which may potentially be because those interventions cannot restore the altered frequency behaviors (e.g., frequency shift and/or temporal dedifferentiation), the important factor underlying the etiology of cognitive impairment. Therefore, age-related declines in brain function can be linked to altered frequency characteristics, which may be preserved by strategies targeting the restoration of such frequency characteristics (i.e., rhythmic brain stimulation techniques).

## Main perspective

Non-invasive rhythmic brain stimulation techniques have been widely used in the rehabilitation of cognitive and brain functions in relatively older adults (Tatti et al., 2016; Assenza et al., 2017), which facilitate the neural entrainment or resonance (Henry et al., 2017) of the brain activities. These techniques are usually imposed physically or psychologically. The former primarily includes repetitive transcranial magnetic stimulation (rTMS), transcranial alternating current stimulation (tACS), and oscillatory transcranial direct current stimulation (O-tDCS) (Wischnewski et al., 2019; Chou et al., 2020; Qiao et al., 2022); and the latter includes cognitive tasks or sensory stimulations focusing on particular frequencies (Norcia et al., 2015; Wang et al., 2016, 2019).

In the application of these techniques, the selection of appropriate stimulation frequency is critical to maximize their benefits. For instance, the effects of 4 and 7 Hz tACS on working memory are quite different, even though they all belong to the theta rhythm (Wolinski et al., 2018). More precise design of targeting frequency in rhythmic stimulation is critical to improve the efficiency of the intervention (Tatti et al., 2016).

**FIGURE 1:**
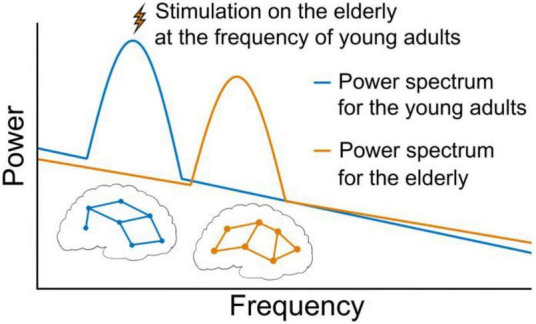
Illustration for the perspective. The overall power and frequency patterns in healthy brain signals follow the scale-free law in which power is proportional to 1/frequency. There are also some oscillations (e.g., the two peaks on the power spectra) of brain signals which correspond to different network patterns (e.g., the two networks in the figure). Due to the shift of frequency between adjacent frequency bands and/or temporal dedifferentiation among multiple bands, the decrease in the efficiency of neural activity and related functional declines are often observed in aging brains. The application of rhythmic brain stimulation to modulate the natural frequency in aging group to that of younger brains may thus help retard the decline of brain functions by reversing neural activities in aging brains to their younger and healthy states.

Natural frequencies of neural activities related to particular cognitive functions are defined as the frequencies at which neural activities and/or cognitive functions can achieve the optimal efficiencies. The optimal efficiencies of neural activities and/or cognitive functions are oftentimes observed in people younger than 40 years old (i.e., young adults) (Edde et al., 2021). For example, the resonant frequencies in Brodmann areas 6, 7, and 19 appeared at about 29, 18.6, and 10.8 Hz, respectively, in people about 30 years old (Rosanova et al., 2009). Another example is that the natural frequency of sustained attention occurred at around 0.05 Hz in children and young adults (Qiao et al., 2022). The resonances at natural frequencies of neural activities (Rosanova et al., 2009) related to sensorimotor or other behavioral performances (Lu et al., 2017; Klimesch, 2018) have been demonstrated. Appropriately characterizing these natural frequencies, which are the promising targets for rhythmic stimulations, is thus important for the design of these techniques. Recently, Qiao et al. (2022) observed that as compared to conventional tDCS, the O-tDCS targeting the potential natural frequency (i.e., 0.05 Hz) of sustained attention may induce significantly greater effects on this function, suggesting that using the natural-frequency-based rhythmic stimulation can further help the higher-order cognitive function. These findings indicate that natural frequencies may serve as precise and efficient targets of rhythmic interventions to improve cognitive and other functions (Lee et al., 2021), holding great promise of retarding the age-related decline of brain functions by reversing frequency shift and temporal dedifferentiation in aging brains (see Figure 1).

## Discussion

Rhythmicity is a critical component for the regulation of neural activities related to cognitive and other important functions. Rhythmic brain stimulation techniques have been demonstrated to improve or restore neural activities at particular frequencies (Klink et al., 2020; Qiao et al., 2022), indicating it may be a novel strategy for the restoration of functions in aging by entraining the altered frequencies in aging brain to those in younger and healthy brain. Still, though it is of great promise, future work is highly demanded and warranted to (1) explicitly examine and characterize the efficacy of this kind of technology, helping maximize its benefits at individual level (Knyazeva et al., 2018; Jafari et al., 2020; Wang et al., 2022); and (2) determine the optimal design of this intervention that would be appropriate for different populations (e.g., those with and without neurodegenerative diseases), including the immediate and longer-term effects, dose-response relationship, etc. (Wang et al., 2015; Solomon et al., 2021).

Taken together, the natural-frequency-based rhythmic brain stimulation techniques hold great potential to restore brain functions in aging process with their significant benefits, which may be expanded to benefit those suffering from neurodegenerative diseases and neuropsychiatric disorders (Mingoia et al., 2013). Future research efforts are warranted to provide critical knowledge and insights into the relationships between natural frequency of brain activities and functional performances, as well as the underlying pathway through which the modulation of such frequency benefits the functions.

## Data availability statement

The original contributions presented in this study are included in the article/supplementary material, further inquiries can be directed to the corresponding authors.

## Author contributions

JQ completed the manuscript writing. SW, YW, and JQ were responsible for the design of the perspective, contributed to the article, and approved the submitted version.
